# Active semi-supervised learning for biological data classification

**DOI:** 10.1371/journal.pone.0237428

**Published:** 2020-08-19

**Authors:** Guilherme Camargo, Pedro H. Bugatti, Priscila T. M. Saito

**Affiliations:** 1 Department of Computing, Federal University of Technology - Paraná, Cornélio Procópio, PR, Brazil; 2 Institute of Computing, University of Campinas, Campinas, SP, Brazil; Korea National University of Transportation, KOREA, REPUBLIC OF

## Abstract

Due to datasets have continuously grown, efforts have been performed in the attempt to solve the problem related to the large amount of unlabeled data in disproportion to the scarcity of labeled data. Another important issue is related to the trade-off between the difficulty in obtaining annotations provided by a specialist and the need for a significant amount of annotated data to obtain a robust classifier. In this context, active learning techniques jointly with semi-supervised learning are interesting. A smaller number of more informative samples previously selected (by the active learning strategy) and labeled by a specialist can propagate the labels to a set of unlabeled data (through the semi-supervised one). However, most of the literature works neglect the need for interactive response times that can be required by certain real applications. We propose a more effective and efficient active semi-supervised learning framework, including a new active learning method. An extensive experimental evaluation was performed in the biological context (using the ALL-AML, Escherichia coli and PlantLeaves II datasets), comparing our proposals with state-of-the-art literature works and different supervised (SVM, RF, OPF) and semi-supervised (YATSI-SVM, YATSI-RF and YATSI-OPF) classifiers. From the obtained results, we can observe the benefits of our framework, which allows the classifier to achieve higher accuracies more quickly with a reduced number of annotated samples. Moreover, the selection criterion adopted by our active learning method, based on diversity and uncertainty, enables the prioritization of the most informative boundary samples for the learning process. We obtained a gain of up to 20% against other learning techniques. The active semi-supervised learning approaches presented a better trade-off (accuracies and competitive and viable computational times) when compared with the active supervised learning ones.

## Introduction

The amount of information available has been increasing, due to new means of acquisition, increased storage capacity and speed of communication, producing large datasets. In this scenario, urgent solutions are needed for the processing, classification and organization of this data, for further manipulation of the dataset. To do so, textual information can be used to annotate these datasets. Although, it has certain practicality and simplicity, manual annotation has several limitations. One of them refers to the impossibility of making complete manual annotations in each sample of the dataset, considering a large application. In addition, the participation of multiple specialists may be required in order to avoid annotation errors. Besides, different individuals can provide different descriptions for the same sample (i.e. the provided information is subjective, generating inconsistencies and unsatisfactory results).

However, the description of the semantic content of the samples by one or more keywords (labels) is still the most efficient and direct form of access to information. In this context, efforts have been made investigating automatic sample annotation techniques [1–6]. These annotation techniques usually require a standard classifier—or collection of classifiers [7, 8]—trained from a dataset that is fully or partially labeled. In the latter case, the *semi-supervised learning* approach can be used [3, 9–16], where the labeled samples (which are almost always scarce in the datasets) can propagate their labels to unlabeled samples (which represent the vast majority of them in the datasets). In the literature, it can be seen that the semi-supervised approach produces considerable improvements in the accuracy of the classifier [17–21]. However, as the datasets are increasing [22], there are two fundamental problems to be solved. One of them is the *reduction* of the dataset in a treatable number of representative samples for the learning. Another one is related to the *selection* of a smaller number of those (from the reduced set) most important for manual annotation and pattern classifier training. Both problems have been successfully addressed using *active learning* techniques [23–32].

In the active learning, the classifier learns at the same time that it selects the training samples and suggests their labels. A specialist participates in its learning process, only correcting or accepting the labels provided by the classifier. The learning process is iterative, until the obtained results are satisfactory. The goal is to minimize specialist involvement without losing control over the classifier learning process. By combining active learning (AL) and semi-supervised learning (SSL) techniques, it would be possible to select the most significant samples from the dataset. They enable to compose the labeled training set and propagate their labels to the unlabeled training set, constructing a more robust classifier. Then, the classifier can be used for automatic annotation of unlabeled samples from other datasets.

Some works have applied the integration of AL and SSL on biological datasets. [33] explored semi-supervised and active learning based on gaussian mixture models for microalgae classification. [34] proposed a logistic regression model combining SSL and AL. They used the unlabeled samples with least cost in an attempt to improve the disease classification. However, their approach requires a previous labeled dataset. Besides that, in their experiments, the authors presented comparisons between only logistic approaches. Another work [35] employs a self-training method in which the entropy of unlabeled samples is used in the active learning process, while the semi-supervised learning uses the probability distribution of all possible labels for the samples. Other combinations of active learning and semi-supervised techniques have been proposed in the literature and somewhat successful when applied to distinct contexts, such as: face recognition [36], diagnosis of intestinal parasites in humans [30], extraction of protein interaction sentences [37], unknown and label-scarce classes [38], sound classification [39], intrusion detection system [40, 41] and textual classification [42].

However, most of them have focused on unfeasible solutions for real-world applications, which may require interactive response times and human intervention, since they neglect the computational cost of the techniques used, and do not consider the selection of the most informative samples for the learning process. Moreover, most works in the literature propose active learning strategies, which perform the classification of all samples of the dataset, followed by the organization and selection of the most informative ones at each learning iteration. When working with real datasets, this process becomes inefficient or impractical to be performed computationally.

### Contributions

This paper proposes a more effective and efficient learning approach to cope with: i) a higher proportion of unlabeled data; ii) scarcity of labeled data; iii) the need for a significant amount of data labeled by a specialist to obtain high accuracies by the classifiers; iv) difficulty in obtaining annotations made by a specialist; v) the need for interactive response times for the learning process. Therefore, the main contributions of this paper includes an active semi-supervised learning framework (FASSL) jointly with a new active learning method, named as Root Distance based Boundary Sampling—RDBS. We accomplished an extensive experimental evaluation in the biological context and the obtained results, comparing with state-of-the-art works, testify the benefits of our RDBS method and our active semi-supervised approach. Through our active semi-supervised learning framework we can significantly improve the learning of the classifier. It is possible to achieve higher accuracies faster by using smaller amounts of samples, learning iterations and interactions with the specialist. The selection criteria adopted by RDBS, based on diversity and uncertainty, allow the prioritization of the most informative boundary samples. Therefore, a smaller number of more informative samples previously selected (by our active learning strategy) and labeled by the specialist can more effectively (i.e. with fewer errors) propagate the labels to a set of unlabeled data (through the semi-supervised strategy). Hence, we do not need that the specialist spends time and effort to label a large dataset.

## 1 Background

### 1.1 Active semi-supervised learning paradigm

In the active semi-supervised learning (ASSL), the training set consists of unlabeled and labeled samples. As aforementioned, since the cost associated with the sample annotation process is high (and it can require the opinion of one or more specialists), the smallest possible set of samples should be labeled. Moreover, it is important that this set contains the most significant samples for the classifier training.

Therefore, active learning strategies can be used to select the most informative samples, which will comprise the labeled part of the training set and then propagate the labels to the samples of the unlabeled part. This is an iterative learning process, in which the classifier participates actively in their learning process along with the adopted selection criteria, helping to choose the most informative (more diverse and more uncertain) samples to be classified. After selection, such samples are displayed to a specialist, who confirms or corrects the labels provided by the classifier. Subsequently, after the confirmation/correction of the labels, these labeled samples are inserted into the training set along with unlabeled samples and a new instance of the semi-supervised classifier can be generated.

**Fig 1:**
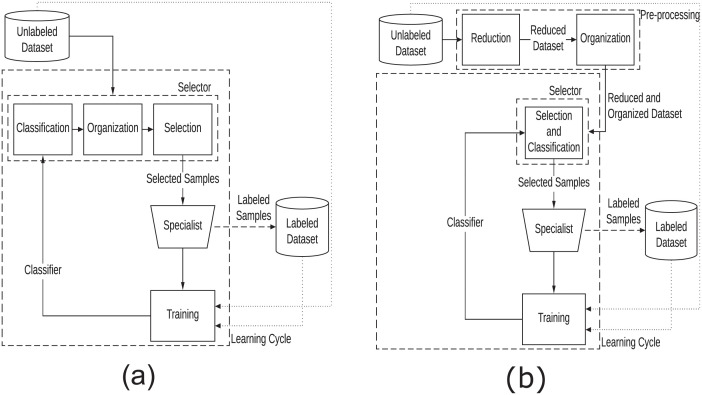
Workflow of the active semi-supervised learning. (a) traditional paradigm. (b) adopted paradigm.

Fig 1 describes the ASSL paradigm, as well as the main differences between the traditional (Fig 1a) and the adopted (Fig 1b) paradigm. In the traditional paradigm (Fig 1a), it is performed the classification and organization of the whole large unlabeled dataset, at each learning cycle. Next, a subset of more informative samples is selected, according to the selection criterion adopted, and analyzed by a specialist. Then, the classifier is obtained from labeled and unlabeled training samples.

Considering real applications and their response time constraints, the use of this ASSL paradigm (Fig 1a) becomes unfeasible, due to the computational times required by the classification, organization and selection processes, considering all the samples of the dataset at each learning iteration.

To solve the unnecessary processing (classification, organization and selection) of the entire dataset throughout the learning iterations, we adopted a more efficient ASSL paradigm (Fig 1b) proposed by [30]. The main difference from the traditional paradigm is that the processes of reduction and organization are performed only once. At each learning iteration, a subset of preorganized samples are selected. Thus, the learning cycle becomes faster, as the reduction and organization processes are performed previously. In addition, the classification process does not involve all samples of the learning set, as in the traditional paradigm. The classification and selection processes are performed alternately, until the desired number of samples is obtained.

In this context, in the selection process, different strategies can be explored to select the most informative samples. Section 1.2 describes the selection strategies considered in our experiments in order to validate our proposed selection strategy (presented in Section 2). Besides that, to the best of our knowledge, these selection strategies were not applied considering the semi-supervised approach and they were not analyzed considering the datasets explored in the present work.

### 1.2 Active learning strategies

Several strategies can be used to select a small set of samples, which must be annotated by a specialist, constituting the labeled part of the training set. We can simply randomly select a number of samples from the learning set and add to the training set, through a *randomized* (Rand) method. However, it is important to select the most informative (diverse and uncertain) samples to obtain a robust classifier more quickly. Some works have used clustering techniques to improve the selection [43–45]. Different clustering techniques can be applied. In our experiments, we considered the *k*-means technique for all methods that require the clustering of the learning samples. In [43] is proposed the *Cluster Rand* (Clu) method, in which the learning samples are grouped into *k* distinct clusters, where *k* is the number of classes. At each iteration, samples are randomly selected from each cluster, trying to get (diverse) samples from different classes.

Recently, more efficient works have been proposed, according to the paradigm aforementioned and presented in Fig 1b. In [44] is proposed the *Increasing Boundary Edges* (IBE) method, which reduces and organizes the learning samples previously. Initially, the learning samples are grouped, obtaining a reduced set formed by samples that represent the roots of the clusters and samples that constitute boundary between distinct clusters. Then, this reduced set is also organized previously. The organization criterion consists in calculating the distances between pairs (edges) of boundary samples followed by ordering them in increasing order of distances. During the learning cycle, samples in the ordered list of edges are analyzed. One edge at a time is obtained and the samples that constitute it are labeled by the current instance of the classifier. If the classifier predicts distinct labels for each sample, they will be selected to be displayed in the next iteration. Otherwise, the next edge in the list is obtained and labeled by the classifier. This process continues until the desired number of samples is obtained. By selecting only the roots and the boundary samples for the learning process, it is expected that the classifier can learn faster compared to the methods that use the entire dataset. The reason for analyzing the boundary samples that are closest to each other is that although these samples belong to distinct clusters, they have a high degree of similarity, due to their smallest distances. Therefore, such samples are the most difficult (uncertain) to be labeled by the classifier and consequently, the most informative ones for its learning.

On the other hand, in [45] is proposed the *Root Distance-based Sampling* (RDS) method, using both diversity and uncertainty criteria to select the most informative samples. The learning samples are also grouped into *k* distinct clusters *C*_*i*_, *i* = Â 1, 2, …, *k*. Next, *k* lists are created, where each list *L*_*i*_ comprises samples belonging to the corresponding cluster *C*_*i*_. Each sample *s* from the list *L*_*i*_ is organized in increasing order of distances, according to the distance between the sample *s* and the root sample *r* that represents the corresponding list *L*_*i*_. The selection criterion consists of obtaining a set of (diverse) samples from each list *L*_*i*_ and prioritizing those more informative (uncertain) samples. Then, the selection is done on each *L*_*i*_, so that the classifier predicts a label on the sample *s* under analysis and compares it with the label of the root sample *r*. If the labels are distinct, the sample *s* is selected; otherwise, the analysis is done with the next sample from the list *L*_*i*_. If all samples in the list *L*_*i*_ are analyzed and the adopted criterion (of different labels) is not satisfied, the sample, referring to the greater distances between it and the root sample *r* that represents the corresponding list *L*_*i*_, is prioritized and selected.

## 2 Proposed framework and implementation

**Fig 2:**
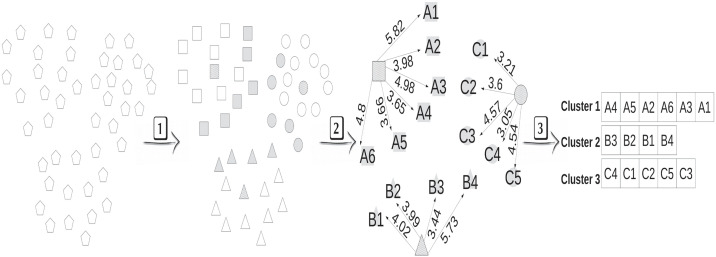
Stages of the RDBS active learning method consisting of the steps: 1) clustering, where root and boundary samples are identified, 2) distances between root and boundary samples from each cluster are calculated and 3) samples are organized into distinct lists in increasing order of distance. Pentagons represent samples before clustering, cracked samples represent root samples and gray samples represent boundary samples.

In this work, we propose an active semi-supervised learning framework (FASSL) https://github.com/btguilherme/FASSL, jointly with a new active learning method, named Root Distance Boundary Sampling (RDBS). The idea consists of a smaller number of more informative samples, previously selected (by the active learning strategy) and labeled by the specialist, can propagate the labels to a set of unlabeled data (through the semi-supervised strategy). The RDBS method (Fig 2) presents a better form of reduction and organization, selecting more relevant samples in relation to those selected by the literature strategies.

Our strategy proposes a reduction process before the organization of the learning samples. RDBS preorganizes the samples and prioritizes the selection of diverse and uncertain samples for the training. Initially, the learning samples (*Z*_2_) are grouped (Step 1) by the clustering technique in *k* clusters *C*_*i*_, *i* = 1, 2, …, *k*, and then *k* distinct lists *L*_*i*_ are created. Each list *L*_*i*_ is composed of boundary samples belonging to the cluster *C*_*i*_, instead of all the samples from the cluster *C*_*i*_.

After the reduction process, which obtains the sets of roots *R* and boundary samples *B* from the clustering, the distances between the root and the boundary samples of the same cluster are calculated (Step 2). Then, the boundary samples are previously organized based on increasing order of these distances (Step 3). At each iteration, samples are selected from each of the lists, in order to obtain diverse samples, from different classes. In addition, each sample obtained from each of the lists is selected only if it presents a label (provided by the current instance of the classifier) different from the label of the corresponding cluster root (in an attempt to select uncertain samples).

**Fig 3:**
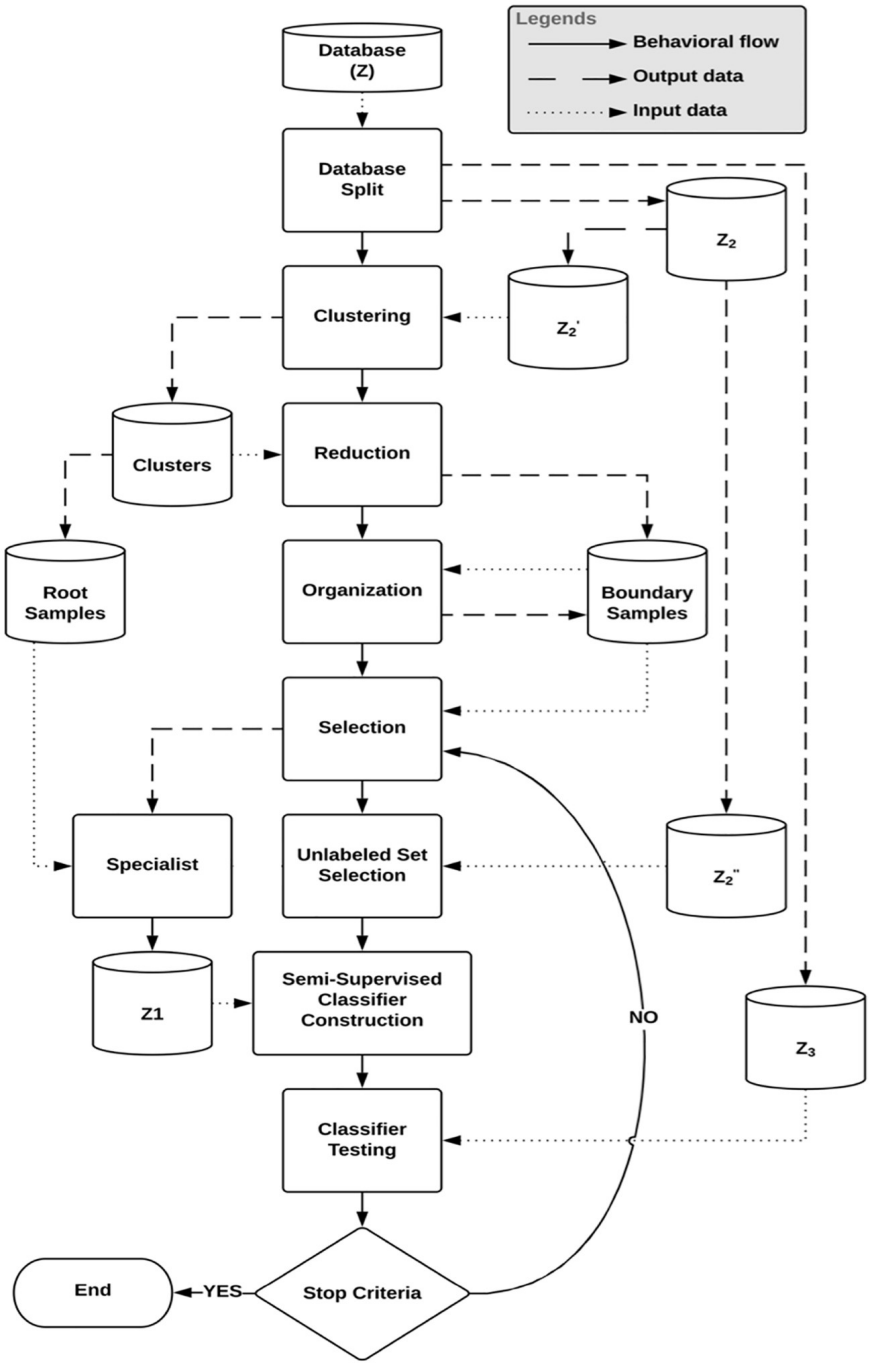
General flowchart of the proposed framework.

By selecting only the boundary samples and the roots in the learning process, it is expected that the classifier will learn faster compared to the techniques that use the entire dataset, since such samples show to be more informative to the classifier learning. The computational processing will be also reduced, because the set of boundary samples is a subset of samples from the learning set (*B* ⊂ *Z*_2_), and generally, *B* ≪ *Z*_2_. Fig 3 illustrates the behavior of the framework, which performs the following processes:

- Dataset split: the division is performed from the dataset *Z* into two subsets, *Z*_2_ and *Z*_3_, representing the learning and test sets, respectively. After the first division of *Z* into *Z*_2_ and *Z*_3_, there is a subdivision of *Z*_2_ into $Z_{2}^{\prime }$ and $Z_{2}^{\prime \prime }$, which represent the learning sets, wherein samples will be selected to compose the labeled and unlabeled training sets, respectively, required by the semi-supervised learning process.
- Clustering: the clustering is performed from $Z_{2}^{{}^{\prime }}$ and it is generated *k* clusters. There are no restrictions, any clustering technique can be applied. Besides, in general, the number of clusters (*k*) is equal to the number of classes (*nc*). However, the number of clusters to be generated may vary, depending on the need and the application. We can generate a larger number of clusters when compared with the number of classes (i.e *k* > *nc*) to select representative samples that cover all/most classes, since the first learning iteration.
- Reduction: the learning samples are previously reduced. From the clustering, it is possible to find the samples that are at the boundary between different clusters. These samples are considered boundary samples if we analyze their *k*-nearest neighbors and there is at least one nearest neighbor that is in a distinct cluster.
- Organization: after the reduction process, the obtained boundary samples are also previously organized. *k* distinct lists *L*_*i*_ are created, *i* = 1, 2, …, *k*. Each list *L*_*i*_ is composed of boundary samples belonging to the corresponding cluster *C*_*i*_. These boundary samples are organized based on increasing order of distances between the root and the boundary samples of the same cluster calculated.
- Selection: the adopted criteria are based on diversity and uncertainty. At each learning iteration, samples are selected from each of the lists *L*_*i*_, in order to obtain diverse samples. Such samples from distinct lists are expected to be samples of distinct classes. Although, there is already a pre-organization of the samples in each of the lists, the classifier participates in its learning process, as it assists in the selection of the most informative (most uncertain) samples. To select the most informative subset of samples from each list, one sample at a time is selected only if it presents a label (provided by the current instance of the classifier) different from the label of the corresponding cluster root. Our strategy does not require the classification of all learning samples at each iteration.
- Semi-supervised classifier construction: in the first iteration, the root samples of each cluster are selected and displayed to the specialist, who will annotate them. Next, to compose the labeled set of the semi-supervised learning, the annotated root samples are added to the training set *Z*_1_. In the subsequent iterations, boundary samples are selected according to the adopted selection criteria and displayed to the specialist, who will confirm or correct the labels provided by the current classifier. Then, these samples are added to the training set *Z*_1_, jointly with the unlabeled sample set (randomly selected from $Z_{2}^{\prime \prime }$), composing the training set *Z*_1_ for the semi-supervised learning.
- Classifier testing: at each new iteration, the constructed classifier can be applied to *Z*_3_, to evaluate its performance in an unknown dataset during its learning process. The learning cycle is repeated until the stop criteria is not satisfied, i.e. all boundary samples are considered or the specialist is satisfied with the classification accuracy obtained.

## 3 Experiments

To validate our framework jointly with our proposed selection strategy, we performed an experimental evaluation considering three public datasets (Section 3.1) and different scenarios (Section 3.2).

### 3.1 Datasets

The *ALL-AML* [46] dataset consist of data from 72 patients presenting distinct types of leukemia. There are 47 patients with acute lymphoblastic leukemia (ALL), characterized by the malignant production of immature lymphocytes in the bone marrow. Also, there are another 25 patients with acute myeloid leukemia (AML), characterized by the rapid proliferation of abnormal and malignant cells—the blasts—that do not mature, do not play their role, and still accumulate in the bone marrow, interfering with the normal production of other blood cells. Each of the 72 patients had bone marrow samples obtained at the time of diagnosis. Furthermore, the observations have been assayed with Affymetrix Hgu6800 chips, resulting in 7, 129 gene expressions (Affymetrix probes) and their composition.

The *Escherichia coli* (or *E.coli*) [47] dataset is public and describes regions of cellular localization of proteins. This dataset is represented by 336 samples, 8 classes and 8 attributes, where each sequence is categorized by its localization site.

The *Pl@antLeaves II* [48] dataset is a subset of the *ImageCLEF2012* [49] dataset, which contains different types of leaves from trees of the Mediterranean region of France. The subset considered in our experiments is composed by 3, 655 scan-like samples from 53 classes (species). To describe these samples, we extract 423 features based on shape, texture and color, where the feature vector consists of: [1–5] scatter measures as a function of signature, [6–13] chain code histogram, [14–139] Fourier descriptors, [140–179] quantified change histogram, [180–231] Haralick descriptors, [232–359] edge and interior histograms generated by BIC descriptor, [360–423] accumulated histogram by CGCH descriptor. The values in brackets represent, respectively, the initial and final positions of a given feature descriptor.

### 3.2 Scenarios

Initially, experiments were performed to evaluate and compare the performance of each supervised and semi-supervised classification strategies using each dataset (ALL-AML, E.coli and Pl@antLeaves II). For the supervised classification, we considered the classifiers: *Random Forest*—RF [50], *Support Vector Machine*—SVM [51] and *Optimum-Path Forests*—OPF [52]. For the semi-supervised classification, we explored the classifier *Yet Another Two Stage Idea*—YATSI [53], which is used jointly with a supervised classifier. Then, we used YATSI with the supervised classifiers RF, SVM and OPF, which were named as YATSI-RF, YATSI-SVM and YATSI-OPF, respectively.

After that, we evaluate the performance of the classifiers (supervised and semi-supervised), with the use of active learning strategies, performing comparisons between the selection strategies (Rand, Clu, IBE, RDS) described in Section 1.2 and our proposed RDBS selection strategy (see Section 2).

We also present comparisons between the traditional (supervised/semi-supervised) approaches and the active (supervised/semi-supervised) approaches to highlight the advantages of the proposed approach. Active supervised/active semi-supervised approaches can obtain high accuracies quickly and with fewer annotated samples.

For all active learning strategies that require the clustering of the learning samples, we considered the *k*-means technique. Other clustering techniques could potentially be employed, however this analysis is not the focus of this work. Then, we define *k* = 2 × *nc* (i.e. the number of clusters is 2 times the number of classes), in an attempt to obtain representative samples that cover all/most classes. If the classifier presents knowledge of all classes since the first iterations, the following iterations are benefited by such information, considering that the classifier cooperates in selecting samples for its own learning.

In our experiments, we compared the performance of each method measuring the accuracy on an unseen test set and the computational time for the training and the classification processes. We also considered the computational time for selecting the most representative samples throughout the learning.

The results reported in Section 2 were compiled from the average of experiments run 10 times, with randomly generated sets of samples for the learning and test sets, for accuracy measures. We chose 80% of the available samples for learning and 20% for testing. To conduct fair comparisons between the learning techniques, we consider the same (10) training and test sets for each of them.

Then, each learning and test sets was applied to the supervised classifiers. For the full operation of semi-supervised classifiers, labeled and unlabeled data sets are required to compose the training set. Therefore, the learning set was subdivided into two subsets, being 50% of the samples for one subset and 50% for another, in which samples will be selected to constitute the labeled and the unlabeled training sets, respectively, as required by the semi-supervised learning process. For fair comparisons, in this case, we also consider the same division and samples for all learning techniques.

To attain unbiased analysis, we considered the size of the selected set of each iteration as being the same for all (supervised and semi-supervised) strategies. Considering the semi-supervised learning, for selection of the labeled part of the training set, we established the number of samples per iteration as 2 times the number of classes. This number of samples is the same number of clusters generated by the clustering technique. To compose the unlabeled sample set, we select half of the size of the labeled sample set.

## 4 Results and discussion

**Fig 4:**
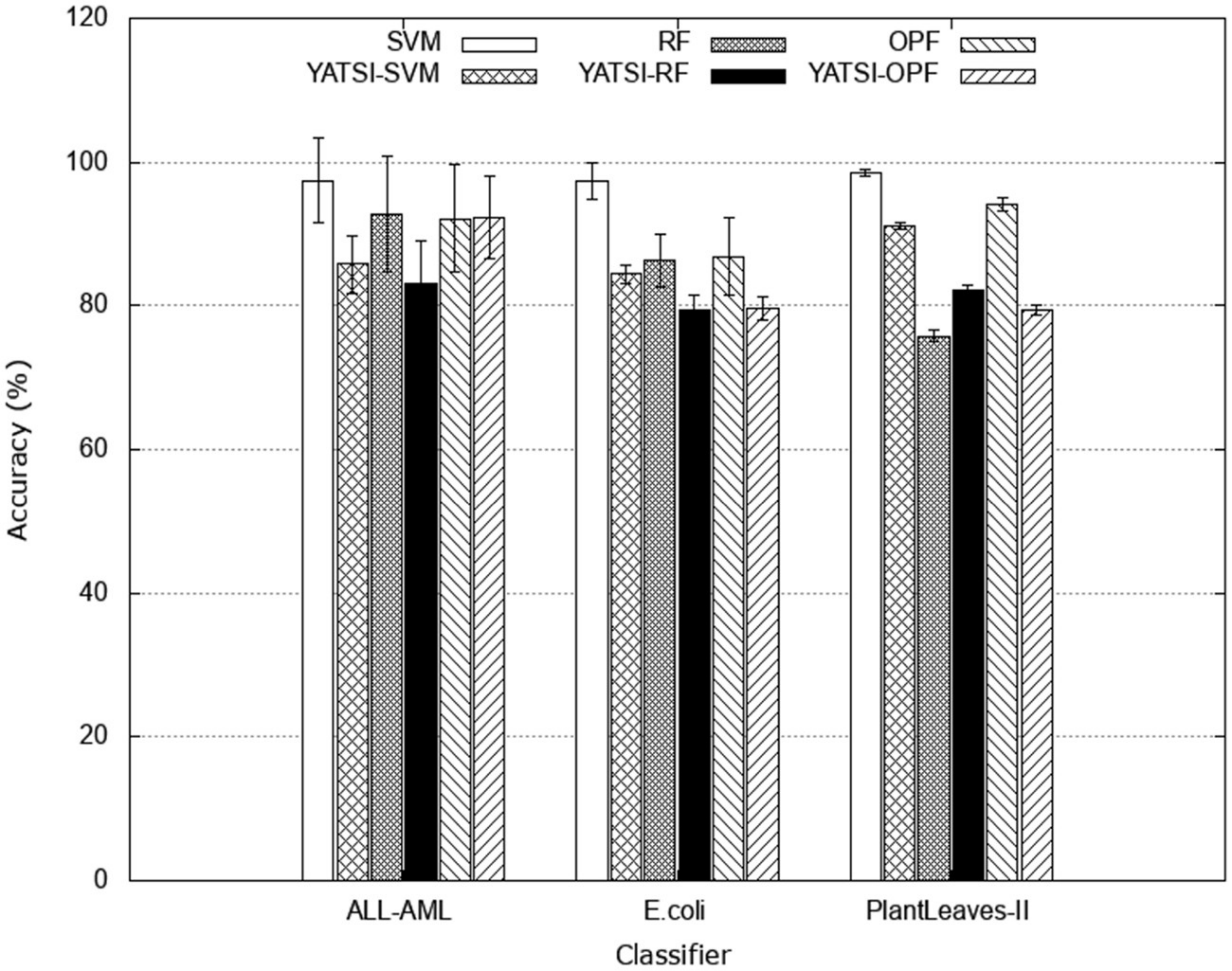
Average accuracies obtained by baselines approaches without considering sample selection strategies, using the *ALL-AML*,*E.coli*, and *Pl@ntLeaves II* datasets and the classifiers (SVM, YATSI-SVM, RF, YATSI-RF, OPF, and YATSI-OPF).

Initially, Fig 4 shows the average accuracy obtained by each baseline approach without considering sample selection strategies for each dataset (ALL-AMl, E.coli, and Pl@ntLeaves II) and classifier (SVM, YATSI-SVM, RF, YATSI-RF, OPF, and YATSI-OPF).

Considering the supervised classifiers, SVM had the highest accuracies. Related to the semi-supervised ones, YATSI-SVM and YATSI-OPF presented the best results. We also compared the performance obtained by the supervised and semi-supervised classifiers. It can be observed that the semi-supervised classifiers presented significant results in relation to the supervised ones, considering that through a semi-supervised approach a small number of annotated samples can propagate the labels to a set of unlabeled samples, reducing the time and effort of the specialist in the annotation process. The semi-supervised classifiers can achieve accuracies similar to those obtained by the supervised ones and with less annotated samples. Note that the semi-supervised approach uses only 50% of annotated samples and 50% of non-annotated samples for training the classifiers.

**Fig 5:**
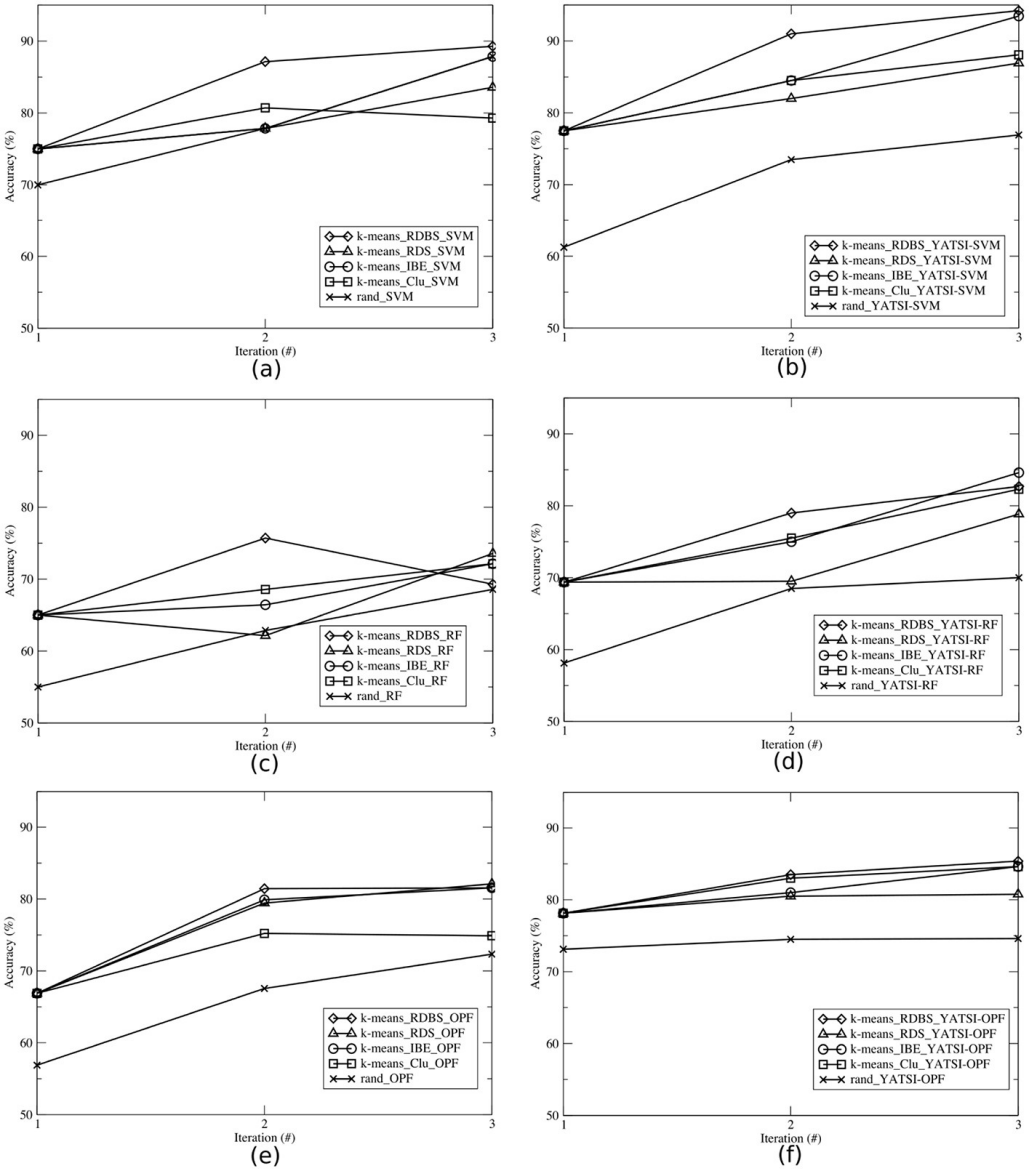
Average accuracies per iteration obtained by each selection strategy (RDBS, RDS, IBE, Clu, and rand), using the *ALL-AML* dataset and the classifiers: (a) SVM, (b) YATSI-SVM, (c) RF, (d) YATSI-RF, (e) OPF, and (f) YATSI-OPF.

**Fig 6:**
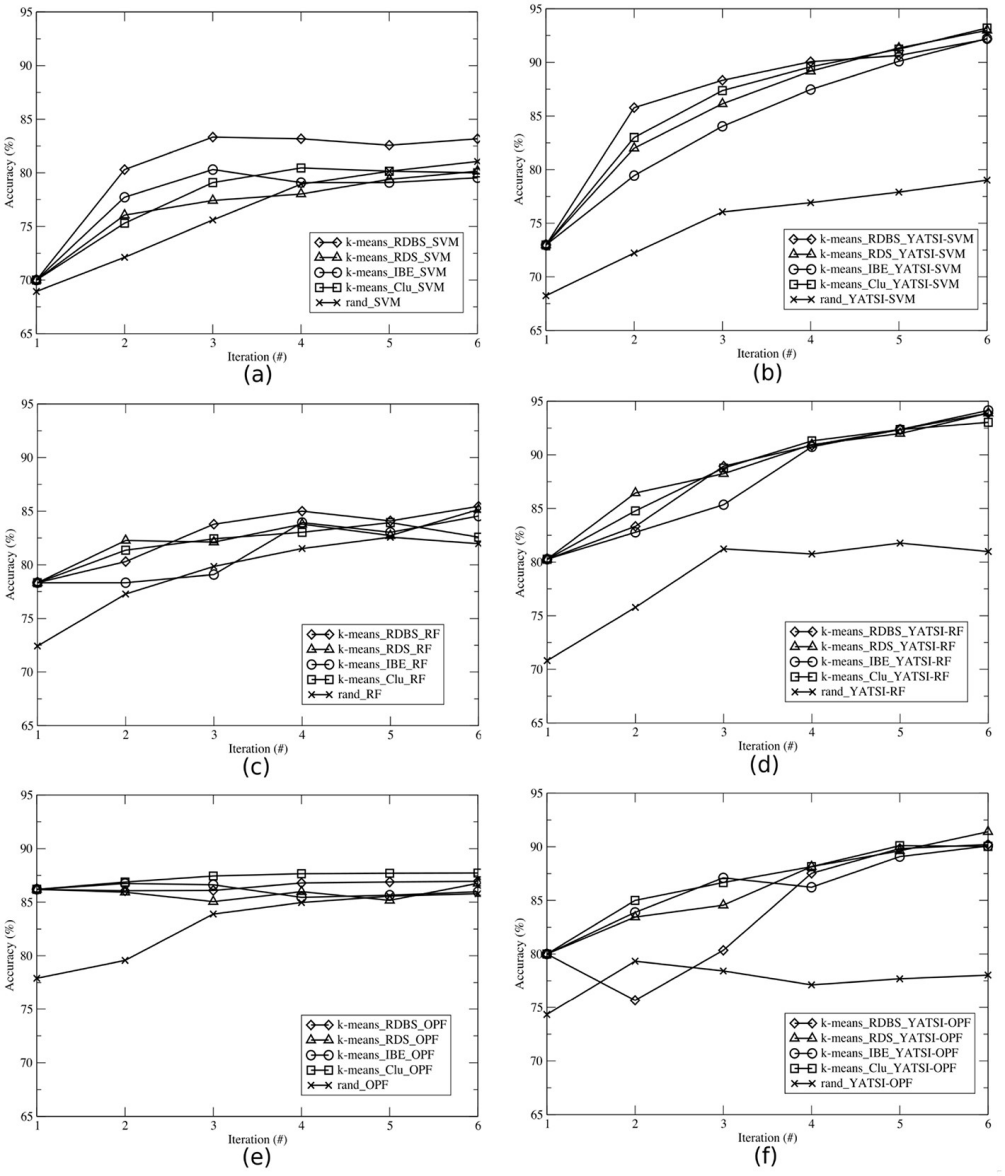
Average accuracies per iteration obtained by each selection strategy (RDBS, RDS, IBE, Clu, and rand), using the *E.coli* dataset and the classifiers: (a) SVM, (b) YATSI-SVM, (c) RF, (d) YATSI-RF, (e) OPF, and (f) YATSI-OPF.

**Fig 7:**
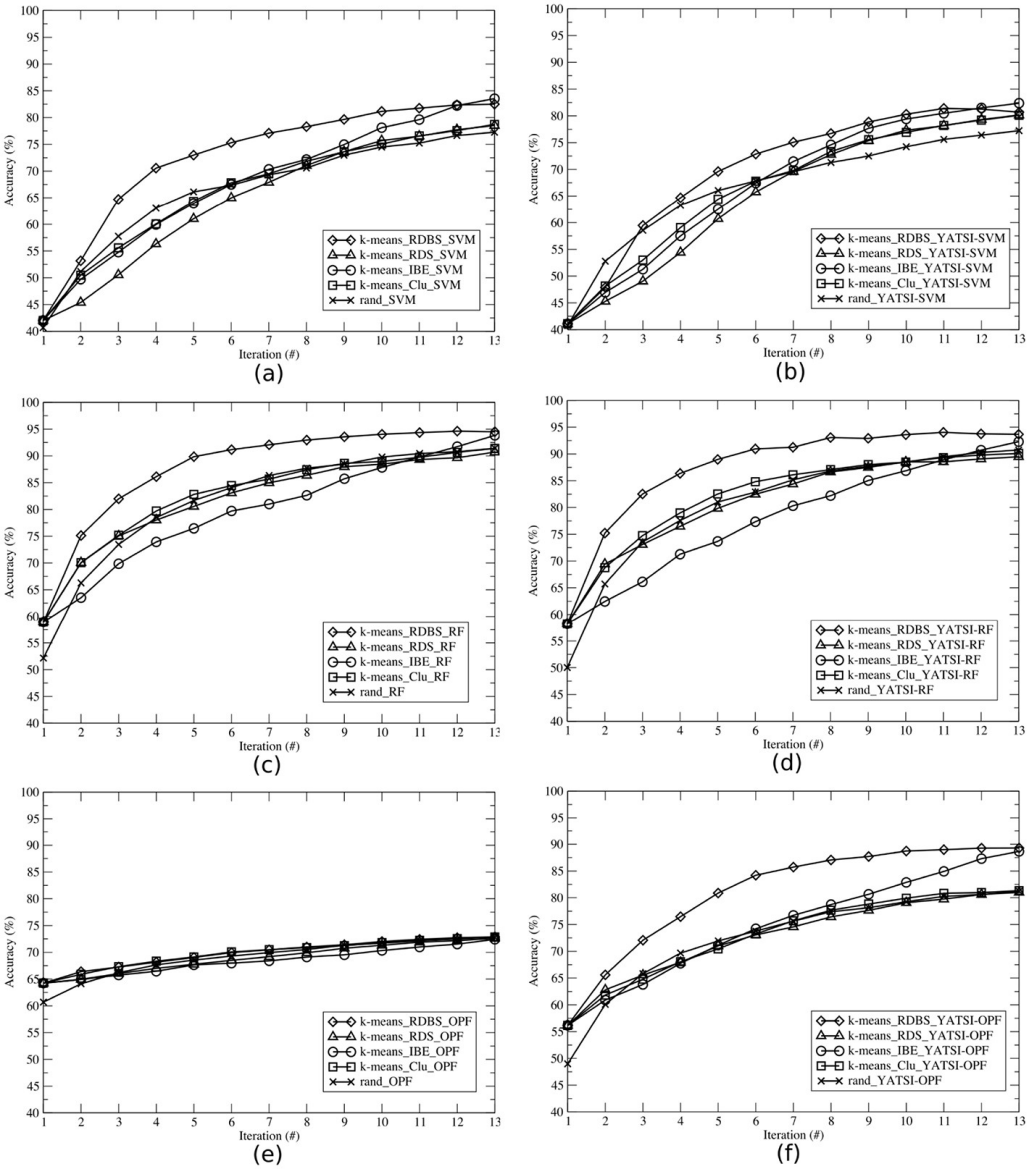
Average accuracies per iteration obtained by each selection strategy (RDBS, RDS, IBE, Clu, and rand), using the *Pl@antLeaves II* dataset and the classifiers: (a) SVM, (b) YATSI-SVM, (c) RF, (d) YATSI-RF, (e) OPF, and (f) YATSI-OPF.

In Figs 5–7, we illustrate the average accuracy per iteration achieved by the learning techniques for the ALL-AML, E.coli and Pl@antLeaves II datasets, respectively. Each learning technique were evaluated considering the supervised (Figs a, c and e) and the semi-supervised (Figs b, d and f) classifiers. To better present the results, each AL technique is represented by a triple, containing the clustering technique, the selection strategy, and the classifier. For instance, *k*-means_RDBS_SVM. The random technique is represented by a pair, which represents the randomized selection and the classifier (e.g. rand_SVM).

Comparing the results of the experiments *with* the application of AL techniques in relation to those obtained by the random technique (i.e. *without* adopting criteria for selection of more informative samples), it is possible to observe (Figs 5–7) that, AL techniques reach a higher average accuracy in the majority of cases. For instance, analyzing the results of Fig 5b, AL techniques presented a gain of up to 20.97% already on the first iteration, against the random technique rand_YATSI-SVM.

Our proposed active learning method RDBS achieved gains in relation to the random techniques for all datasets and (supervised and semi-supervised) classifiers. It is possible to observe that RDBS, in overall, overcomes the other active learning techniques (RDS, IBE, Clu). For instance, our proposed active learning technique, *k*-means_RDBS_SVM, also reached gains of up to 7.63%, 6.64% and 5.17%, when compared with the other active learning techniques (*k*-means_RDS_SVM, *k*-means_Clu_SVM and *k*-means_IBE_SVM, respectively).

For all datasets (Figs 5–7), comparing both supervised (Figs a, c and e) and semi-supervised (Figs b, d and f) approaches, we can note that the semi-supervised presented notable results. Considering the ALL-AML dataset, using the RF classifier, we achieved gains of up to 19.35% with the semi-supervised approach (Fig 5d) in comparison with the supervised one (Fig 5c). The same behavior can be observed for the other datasets and classifiers. For instance, for the E.coli dataset, considering the SVM classifier (Fig 6a and 6b), the AL semi-supervised techniques presented better results in all iterations, reaching a gain of up to 16.51% against the AL supervised ones. For the Pl@antLeaves II dataset, the OPF classifier achieved gains up to 23.21% with YATSI-OPF (see Fig 7e and 7f).

Thus, it is also possible to corroborate for the active learning approaches, considering the final average accuracy, that the semi-supervised classifiers present advantages over the supervised ones, showing higher final average accuracies. Moreover, our new active learning method (RDBS), in most of the scenarios, presents the best results.

We also performed a comparative study between the active learning and the traditional learning approaches. For example, analyzing Fig 4, from the traditional learning, the semi-supervised classifiers reached accuracies about 80% for all datasets. While, the active learning achieved equivalent accuracy, in general, already in the second iteration (see Figs 5–7). Note that, active semi-supervised learning approaches select, at each iteration, the number of samples as 2 times the number of classes to compose the labeled part of the training set and half of the size of the labeled sample set to compose the unlabeled part of the training set. In this context, for the ALL-AML dataset, as *nc* = 2, considering the active semi-supervised approach, we select at each iteration 4 samples to compose the labeled training set and 2 samples to compose the unlabeled training set. It results in 12 samples selected for annotation and to compose the training set in the 2^nd^ iteration. For the traditional learning, it was necessary to annotate all training samples of the dataset. Therefore, we not only reached equivalent accuracies, but also obtained a reduction of 80% of labeled samples used in the learning process. To achieve the accuracies presented in Fig 4, the classifiers used all the samples (i.e. 80% available for training) from the dataset (Table 1), and all of them had to be labeled. On the other hand, active learning approaches aim to achieving higher accuracies faster by using smaller amounts of samples, learning iterations and interactions with the specialist.

**Table 1:** Description of the datasets, including number of samples, classes and features.

	Samples	Classes	Features
ALL-AML	72	2	7,129
E.coli	336	8	8
Pl@antLeaves II	3,655	53	423

In addition to the classification accuracies, another important quality measure is related to the time costs. We present the average computational times in the first three iterations of the training and testing processes, presented by the supervised and semi-supervised classifiers, respectively, for the ALL-AML (Tables 2 and 3), E.coli (Tables 4 and 5) and Pl@antLeaves II datasets (Tables 6 and 7).

**Table 2:** Average computational times and standard deviations (in milliseconds) per iteration for the training and testing processes, using the supervised classifiers for the *ALL-AML* dataset. The best results (shorter times) are highlighted in bold.

	Iteration (#)	Classifier		
		SVM	RF	OPF
Train	1	0.996 ± 0.812	31.476 ± 2.115	**0.016 ± 0.004**
	2	1.032 ± 0.519	33.376 ± 3.370	**0.062 ± 0.008**
	3	1.191 ± 0.260	33.736 ± 3.063	**0.158 ± 0.010**
Test	1	0.308 ± 0.248	**0.066 ± 0.038**	**0.061 ± 0.013**
	2	0.209 ± 0.031	**0.065 ± 0.025**	0.118 ± 0.026
	3	0.201 ± 0.021	**0.061 ± 0.010**	0.131 ± 0.022

**Table 3:** Average computational times and standard deviations (in milliseconds) per iteration for the training and testing processes, using the semi-supervised classifiers for the *ALL-AML* dataset. The best results (shorter times) are highlighted in bold.

	Iteration (#)	Classifier		
		YATSI-SVM	YATSI-RF	YATSI-OPF
Train	1	**2.747 ± 1.765**	32.985 ± 2.587	**2.400 ± 0.814**
	2	**3.296 ± 1.476**	34.066 ± 1.884	**4.514 ± 1.144**
	3	**4.065 ± 1.342**	34.718 ± 2.558	7.535 ± 0.981
Test	1	0.430 ± 0.360	**0.113 ± 0.150**	1.178 ± 0.285
	2	0.424 ± 0.137	**0.192 ± 0.042**	2.495 ± 0.512
	3	0.562 ± 0.140	**0.198 ± 0.019**	4.420 ± 0.651

**Table 4:** Average computational times and standard deviations (in milliseconds) per iteration for the training and testing processes, using the supervised classifiers for the *E.coli* dataset. The best results (shorter times) are highlighted in bold.

	Iteration (#)	Classifier		
		SVM	RF	OPF
Train	1	6.128 ± 1.307	0.679 ± 0.427	**0.007 ± 0.002**
	2	7.262 ± 1.347	0.993 ± 0.132	**0.021 ± 0.005**
	3	7.890 ± 1.372	1.321 ± 0.199	**0.040 ± 0.002**
Test	1	**0.024 ± 0.005**	0.197 ± 0.183	**0.017 ± 0.004**
	2	**0.025 ± 0.005**	0.231 ± 0.098	**0.030 ± 0.004**
	3	**0.029 ± 0.016**	0.200 ± 0.026	**0.044 ± 0.004**

**Table 5:** Average computational times and standard deviations (in milliseconds) per iteration for the training and testing processes, using the semi-supervised classifiers for the *E.coli* dataset. The best results (shorter times) are highlighted in bold.

	Iteration (#)	Classifier		
		YATSI-SVM	YATSI-RF	YATSI-OPF
Train	1	5.982 ± 1.593	1.427 ± 1.553	**0.265 ± 0.451**
	2	8.162 ± 1.704	1.356 ± 0.485	**0.194 ± 0.118**
	3	9.264 ± 2.101	1.705 ± 0.445	**0.334 ± 0.285**
Test	1	**0.083 ± 0.161**	0.253 ± 0.216	**0.107 ± 0.198**
	2	**0.043 ± 0.036**	0.245 ± 0.069	**0.073 ± 0.048**
	3	**0.052 ± 0.038**	0.287 ± 0.055	**0.110 ± 0.055**

**Table 6:** Average computational times and standard deviations (in milliseconds) per iteration for the training and testing processes, using the supervised classifiers for the *Pl@antLeaves II* dataset. The best results (shorter times) are highlighted in bold.

	Iteration (#)	Classifier		
		SVM	RF	OPF
Train	1	397.203 ± 25.948	20.748 ± 2.059	**0.247 ± 0.035**
	2	449.096 ± 11.862	39.960 ± 0.686	**0.942 ± 0.130**
	3	461.495 ± 7.905	60.156 ± 0.529	**2.090 ± 0.155**
Test	1	16.672 ± 1.072	4.829 ± 0.792	**2.194 ± 0.117**
	2	17.605 ± 0.566	4.805 ± 0.396	**3.952 ± 0.095**
	3	17.485 ± 0.392	**5.023 ± 0.319**	5.722 ± 0.069

**Table 7:** Average computational times and standard deviations (in milliseconds) per iteration for the training and testing processes, using the semi-supervised classifiers for the *Pl@antLeaves II* dataset. The best results (shorter times) are highlighted in bold.

	Iteration (#)	Classifier		
		YATSI-SVM	YATSI-RF	YATSI-OPF
Train	1	419.558 ± 32.081	29.019 ± 5.909	**12.596 ± 1.564**
	2	470.855 ± 18.487	49.872 ± 3.763	**27.583 ± 2.151**
	3	486.337 ± 10.488	72.289 ± 3.256	**48.141 ± 2.903**
Test	1	17.747 ± 1.640	**5.109 ± 1.135**	9.876 ± 1.894
	2	21.127 ± 0.693	**6.230 ± 0.672**	19.885 ± 1.342
	3	24.924 ± 0.677	**7.262 ± 1.195**	33.864 ± 1.430

Comparing the supervised classifiers, we can note that OPF presented the best results, regarding the training process, for all datasets (see Tables 2, 4 and 6). For instance, OPF is 7.54 and 213.52 times faster than SVM and RF, respectively (Table 2). Analyzing the test process, RF also presented good results. It is about 3.09 and 2.02 times faster than SVM and OPF, respectively, for the ALL-AML dataset. However, it is possible to note that the OPF classifier, which obtained best performance in the training process, also reached good results for the test time (considering all datasets).

Regarding the semi-supervised classifiers (Tables 3, 5 and 7), the same behavior was verified. For instance, in the E.coli dataset (Table 5), YATSI-OPF is 12.6 and 4.3 times better than YATSI-SVM and YATSI-RF, respectively, in the training process. Considering the testing costs, in general (for the ALL-AML and Pl@antLeaves II datasets), YATSI-RF presented the best results (e.g. 14.10 and 2.87 times faster than YATSI-SVM and YATSI-OPF, respectively, for the Pl@antLeaves II dataset, Table 7). For the E.coli dataset, YATSI-RF and YATSI-OPF presented equivalent results (see Table 5).

Analyzing the time costs between the supervised (Tables 2, 4 and 6) and the semi-supervised (Tables 3, 5 and 7) approaches, in some cases, they are statistically equivalents or the supervised approaches are faster than the semi-supervised ones. Despite these results, we can notice that the semi-supervised approaches present a better cost benefit. For instance, they reached an accuracy gain of 25% against the supervised approaches (see Fig 7e and 7f).

It is also important to analyze the time costs presented by the AL techniques in the selection process. Table 8 summarizes the average accuracies and computational times (for the training, testing and selection processes) obtained by each selection strategy in the 3rd iteration, using the YATSI-OPF classifier for all datasets. We can see that RDBS provides a better tradeoff, i.e. besides the best accuracies, in general, RDBS also presented the best time costs for all three datasets.

**Table 8:** Average accuracies and computational times (in milliseconds) for the training, testing and selection processes obtained by each selection strategy in the 3rd iteration, using the YATSI-OPF classifier for all datasets.

Datasets	Strategies	Accuracies	Train	Test	Selection
ALL-AML	RDBS	85.385	0.075	0.042	0.07851
	RDS	80.769	0.072	0.042	1.83808
	IBE	84.615	0.079	0.047	0.00003
	Clu	84.615	0.076	0.045	1.28907
E.coli	RDBS	80.351	0.005	0.001	0.00720
	RDS	84.561	0.002	0.001	0.03395
	IBE	87.105	0.005	0.002	0.00003
	Clu	86.667	0.002	0.001	0.02442
Pl@ntLeaves II	RDBS	72.073	0.472	0.351	3.64425
	RDS	65.566	0.475	0.319	21.88268
	IBE	63.810	0.528	0.373	0.00011
	Clu	64.798	0.450	0.312	13.59324

In order to validate our proposed framework, we also present comparisons with a recent state-of-the-art active semi-supervised framework [35], considering the RF classifier for other biological datasets, such as Haberman, Heart Statlog and Lymphograph [54] (see Table 9). Our framework achieves higher accuracies and requires fewer (much less than 10% of the dataset of) labeled training samples compared to the state of the art one, considering all datasets and active learning strategies. Such results constitute a valuable contribution as it speeds up the learning process and minimizes the interaction of the specialist in the annotation process.

**Table 9:** Comparisons between our proposed and state-of-the-art active semi-supervised frameworks, considering the RF classifier for other biological datasets. For our framework, the values represent the accuracies and the percentages of labeled samples obtained by each selection strategy (note that we consider much less than 10% of the datasets). For the state-of-the-art framework, the values represent the accuracy obtained with 10% of the datasets.

Datasets	Proposed				State-of-the-art [35]
	RDBS	RDS	IBE	Clu	
Haberman	65.333 (9.8%)	66.000 (9.8%)	72.000 (4.9%)	66.000 (2.4%)	65.656 (10%)
Heart Statlog	76.667 (2.7%)	79.259 (3.7%)	77.222 (5.5%)	76.666 (3.7%)	75.185 (10%)
Lymphograph	71.538 (6.7%)	70.384 (3.3%)	69.615 (3.3%)	68.461 (3.3%)	67.571 (10%)

Thus, the experiments testify that the proposed framework achieved significant gains, not only regarding accuracy, but also w.r.t. computational time costs and specialist’s efforts. It leads to a better effectiveness and efficiency, opening new ways of improvement of the classification process in different contexts.

## 5 Conclusion

In the present paper, we proposed a more effective and efficient learning framework (FASSL), and a new active learning method (RDBS). To validate them, an extensive study and experimental evaluation were carried out taking biological context as focus, presenting comparisons between our proposals and other state-of-the-art learning strategies.

Experiments were performed with the *ALL-AML*, *E.coli* and *Pl@antLeaves II* datasets. Through the obtained results, it can be seen that with our RDBS AL method, the classifier achieves higher accuracies with a reduced number of labeled samples. The adopted selection criteria, based on diversity and uncertainty, allows the prioritization of the most informative boundary samples, reducing the learning iterations and the specialist annotation effort.

Besides, AL techniques using semi-supervised classifiers achieved excellent results. They obtained better accuracies, since the first learning iterations of the classifier, when compared to a randomized method (without AL) using the same classifiers. This same behavior was noted using the supervised classifiers.

Then, although semi-supervised classifiers require more (computational time) processing to the training and testing processes in comparison with the supervised ones, active semi-supervised learning approaches present a better trade-off (competitive and viable times) than active supervised ones.

It is also important to note that our framework can be straightforwardly applied to different contexts, and it is easily extended.
